# Workhouse Diets

**Published:** 1900-10-27

**Authors:** 


### The Institutional Workshop.

### WORKHOUSE DIETS.

The Local Government Board has at last dealt in a fairly complete manner with the workhouse dietaries, and has issued a general order to all boards of guardians in England and Wales, together with an explanatory circular, in which the most minute and careful directions are given as to the apportioning of the various kinds of food. There is at first sight a dash of the humorous in the thought of grave public officials in high position in the public service issuing as a serious public document, with all the solemnity appropriate to a Local Government Board Order, a memorandum filled with petty details as o the carving of meat and the serving of vegetables, as to the necessity of hot plates for hot food, and as to the advantage of remembering that all children's appetites are not the same; and the *Times*, true representative of public feeling, has been moved to mirth thereby. It must not, however, be forgotten that this document, with all its curious provisions as to food, is really the charter upon which depends much of the happiness of nearly a quarter of a million of people whose daily food is to be regulated according to its provisions, and much of the health and future stamina of an enormous number of children, who, from no fault of their own, are thrown upon the rates, and have to depend in regard to every detail of their none too happy little lives upon the intelligence and the goodwill of the guardians of the poor. The question of bread, for example, has long been a cause of friction between certain boards of guardians and the central authority. To many people it has seemed easy to make fun about the regulations in deference to which each pauper, quite regardless of his appetite, had to receive his full allowance of what, in some workhouses, was truly the staff of life. No doubt in many cases these regulations led to endless waste. The bread was not allowed to be served up another day: all that was not eaten had to go to the pig-tub, and thus it came about that an almost necessary link grew up between pigs and paupers. Nevertheless, this little clause, enforcing the full loaf has been, in many workhouses and for many years, the pauper's charter, giving him, in spite of all that parsimonious guardians might do, at least a full supply of his daily bread. Guardians differ much in different parts of the country. In many the old order—the guardians of the public purse—are dying out, and the new order—the real guardians of the poor—are becoming every day more manifest in good works. But there is still a strong leaven of bumbledom in workhouse management, a fact of which the Local Government Board are fully cognisant. Thus it will be noted that in the new order, although the bread may now be so served that each pauper gets at first only a portion of his allowance, helping himself afterwards to more, it is still compulsory that the total amount of bread prescribed by the dietary shall be taken into each dining-room, and that no portion of it shall be removed until the conclusion of the meal. The right also of the pauper to his full allowance of bread or other food, and his right to have it weighed under certain regulations, remain untouched. The order is very carefully and thoughtfully drawn up so as to grant considerable latitude to the guardians, and to enable them to give variety without causing waste. But care is taken that the new regulations shall not be so used as to deprive the pauper of his full supply of food.

Coming now to particulars, it is to be noted that the order will come into force generally from March 25th, 1901, but, at the option of the guardians, may be brought into operation in any union from December 25th next.

The dietaries are to be framed by the guardians in consultation with the medical officer for the workhouse, and before they are finally adopted, they are to be referred in their entirety to the medical officer for a written report upon them, which the guardians must consider; but the function of the medical officer in this respect will be purely advisory, the final decision, and we may add responsibility, resting with the guardians alone, and when these dietaries have been fixed, all inmates, with the exception of the sick, of children under three years of age, and of certain other special cases, must be dieted in accordance with them.

For adult inmates the dietary tables will be for each sex respectively two in number, with certain subdivisions, one of these diets being termed "Plain Diet," and the other, which is to be so framed as to be suitable for infirm but otherwise healthy inmates, being termed "Infirm Diet."

An examination of the order will show that in its application in any workhouse the justice and utility of the new dietaries will depend to a large extent upon the care and completeness with which the classification of the inmates is carried out.

In regard to this classification it is to be noted that the diets to be arranged by the guardians do not apply at all to the sick, properly so called, those that is, who are "dieted under medical supervision," nor to any infirmary or school under administration separate from the workhouse, nor to any casual ward. But in regard to those to whom it does apply the inmates will be divided into six classes and certain sub-classes.

Class 1 and class 2 are males, on plain and on infirm diet respectively; class 3 and class 4 are females on plain and infirm diet respectively; while class 5 and class 6 are children, and class 7, children specially dieted by the doctor.

Taking, then, as a basis these plain and infirm diets for adult, certain additions may be made to them. Thus, whether an inmate be, say, in Class 1, with plain diet, or in Class 2, with infirm diet, the guardians may place him in a sub-class (1A, for example, or 2A), which will give him additional food at lunch or as the guardians may direct. There are a considerable number of sub-classes, and if this classification is carefully carried out, it will give great power of varying the diet according to requirements. For example, it is one of the principles of the poor law that the inmates of the workhouse shall be kept employed according to their capacity and ability, while another principle is that no inmate shall receive any compensation for his labour. Strictly interpreted this not only prevents any money payment to paupers, but prevents their being paid for extra services by extra food. But it is obvious that where extra labour is demanded extra food should be given, not as a payment, but to enable the extra labour to be performed, and the system of sub-classes enables the guardians to do this with great facility. As to the sort of labour which shall be taken as requiring such extra diet this is left to the guardians to decide, but for their guidance the Local Government Board say that they are advised that inmates doing an average day's work (for a healthy able-bodied man of average weight (150 to 160lbs.) 300 "foot-tons," equivalent to a walk of about 16 miles on the level at the rate of three miles an hour, is usually regarded as the mechanical equivalent of an "average day's work") with sustained exertion—e.g., corn-grinding, pumping, stone-breaking or crushing, shifting heavy goods, digging, scrubbing, washing, ironing, &c., should be placed in class 1A men, 3A women for diet; while those employed without sustained exertion—e.g., wood-chopping and wood-bundling, hoeing or weeding, sorting light articles, sewing, &c., should be classed as 1 or 2A men, 3 or 4A women.

The provision in the orders in force for the government of workhouses, whereby guardians are empowered to make extra allowances of food to paupers employed in the household work, is in effect rescinded by the present order.

The key to the proper distribution of the diets will be the proper classification of the inmates, and in regard to this it is pointed out that much assistance will be derived from the examination of each inmate by the medical officer, and we cannot but feel that in actual working it will be found that a considerable amount of somewhat thankless work will be thrown upon the doctors.

The choice of diets is a wide one for all classes, but the use of the sub-classes is intended more for the variation of the "infirm" and "children's" than of the "plain" diets. We may be sure, however, that the scramble among the able-bodied inmates to get on sub-class diet will cause much trouble to individual officers.

For the feeble inmates, however, we can have no doubt at all that the latitude allowed in fixing the diet scale will be of immense advantage, it being possible by aid of the "special infirm diets" to relieve considerably the monotony of workhouse food. To the administration also these diets will give much relief by lessening the crowding of the infirmaries, for many cases which are now sent into hospital merely for the sake of sick diet will now be able to receive what they want in the infirm wards. The feeding of the sick will remain as heretofore the duty of the medical officer, and he will be required to frame suitable scales of sick diet accordingly, in regard to which many useful provisions are laid down.

Take it altogether this elaborate order is likely to be of great benefit to the inmates of our workhouses, and in large institutions will much facilitate administration.

